# Editorial: Where Is Waldo: Contextualizing the Endothelial Cell in the Era of Precision Biology

**DOI:** 10.3389/fcvm.2020.00127

**Published:** 2020-07-31

**Authors:** Zorina S. Galis

**Affiliations:** National Heart Lung and Blood Institute, National Institutes of Health, Bethesda, MD, United States

**Keywords:** endothelial cell, endothelium, single cell analysis, endothelial cell heterogeneity, precision biology

Many readers may recall Waldo, the North American version of the original character of “*Where is Wally?”* introduced in 1987 by British author Martin Handford. The book challenged readers to identify—in post-cards depicting crowded scenes in various locales—a solitary, distinctively equipped world traveler carrying situationally appropriate implements: a walking stick, camera, or skis. As readers of the book “sequels” became more astute, Waldo was recognizable in the context of ever more complex and grander scenes.

## Like Waldo, the Endothelial Cell (EC) Is Hiding in Plain Sight Everywhere

When viewed in various histological “landscapes” ECs may appear sparse due to their peculiar organizing principle into a single cell-thin layer, the endothelium, the contiguous inner lining of all blood and lymphatic vessels, large and small, distributed everywhere in the body. Covering thousands of square meters, ECs, the third most abundant human cell type (1), strategically interface with all tissues, acting as master body-wide integrators and local mediators of tissue survival and function (2). To this day, the endothelium escapes clinical detection, the “out of sight, out of mind” challenge is a likely reason for still underestimating the EC's importance in health and disease. Definitive proof of the EC's very existence had depended on reaching single cell (SC) level resolution through Electron Microscopy (EM) and the discovery of the pan-EC-specific organelle, the Weibel-Palade body (3). Recent SC technologies extract multi-dimensional information about sparse cells in complex mixtures, thus ready to tackle the EC “scarcity” challenge.

## As Observers Have Grown Astute, Just Like Waldo, Ecs Become Recognizable in Ever More Complex Scenarios

The EC makes frequent, yet diverse appearances in different tissue contexts. Extreme examples of EC specialization are found in the key vascular-functional units of different organs, e.g., the “continuous” EC essential for the tight blood-brain barrier vs. the “fenestrated” EC essential for the kidney's filtration function.

Knowledge about ECs has grown through many different types of inquiry. Some investigations have provided “wide and shallow” information, e.g., averaging responses from bulk, mixed populations of ECs cultured *in vitro* or harvested *in situ*. Early “narrow and deep” EM interrogations of ECs reporting context-dependent structural and functional heterogeneity (4) were initially questioned as new technology artifacts. While EC heterogeneity is currently believed essential for the function *or dysfunction* of every tissue (5), the dichotomy between ECs having both body-wide shared and distinctive local variations of structural-functional characteristics remains challenging. The new SC technologies summarized by Chavkin and Hirschi, probing both deeply and widely to extract single EC comprehensive profiles from among more abundant cell assortments, are overcoming both the “sparse” and the “diverse” EC appearances. They should permit us to recapitulate, reconsider, and add new dimensions to the known EC biology. Indeed, Xiang et al. and Feng et al. unravel ECs' exquisite local adaptations based on vessel type or tissue context. Computational tools comparing molecular repertories gathered by various SC technologies and across species, such as those described by Chavkin and Hirschi and Xiang et al., will increase recognition of key molecular features of the EC's functional heterogeneity.

## Like Waldo, Ecs Hold Important Contextual Information, and Reveal Their Precise Location

When Waldo is seen carrying his skis, snow is likely close by! Likewise, exquisitely equipped to adapt, in space and in time, to support each tissue's metabolic and functional needs, ECs have inextricable relations to their natural context and are sensitive reporters of neighborhood's workings, metabolic state, and stresses (6) and Xiang et al., and signal transition to dysfunction or aging (7). Precise characterization of the human EC repertoire central to specialized vascular functional units of different organs, including the kidney, brain, lung, or muscle, stands to be of special translational and clinical value.

The potential of using vasculature as a road map to report the location of every SC in the body is described by Weber et al. The concept is based on the known oxygen-dependence of every mammalian cell, thus its proximity to ECs, and known organ-specific vascular hierarchies. The vasculature provides a built-in solution for a Common Coordinate Framework (CCF), a central challenge to developing high-resolution maps of tissues, organs, or the entire body.

ECs' expression of positional information is already used by cancer therapeutic agents delivered to organ-specific EC “zip codes” (8), identified by phage display (9). Ever more precise EC positional information derived from SC analyses suggests they are fitting “cellular landmarks” for a GPS-like precise positioning when developing SC-resolution tissue maps, even if sites of sampling were unknown. For instance, individual ECs were placed along the brain vasculature, while walking on the endothelium from the arterial to venous side via capillaries (10), and within specific zones of the kidney (6) or the lymph nodes by Xiang et al. The EC's closest cellular neighbors may be identifiable by exploiting molecular fingerprints of their interactions (11). We hypothesize that positioning of other SC within the vital <100 microns radius 3D-map centered around a recognizable EC “zip code” may be inferred from the SC expression of hypoxia-driven gene gradients.

## Precision Biology and the EC

The new era of “Precision Biology,” combining high content investigative technologies and computational analyses including SC methods, reveals comprehensive cellular profiles in their natural contexts and in action, allowing researchers to overcome the “scarcity” and “heterogeneity” challenges specific to cells such as the EC. Zeroing in on the ECs—apparently sparse yet omnipresent, recognizable yet diverse—through the use of SC methods will pay off in essential knowledge about their cellular neighborhoods, including revealing the underpinnings and states of key functional vascular-tissue units specific to different organs such as the brain, kidney, or heart. Precision EC biology will contribute to the development of a variety of new targeted clinical diagnostics and interventions, “Waldo's” important contribution to the future of precision medicine.

## Author Contributions

ZG has conceived and written the editorial and is solely responsible for the content of this article.

## Conflict of Interest

ZG is a US Federal employee. The author declares that the research was conducted in the absence of any commercial or financial relationships that could be construed as a potential conflict of interest.

## Abbreviations

EC: endothelial cell
SC: single cell
EM: electron microscopy
CCF: common coordinate framework.
